# A shared-aperture pentaband antenna with high impedance surface for CubeSat application

**DOI:** 10.1038/s41598-024-66632-7

**Published:** 2024-07-12

**Authors:** Md Nazim Uddin, Elias A. Alwan

**Affiliations:** Department of Electrical and Computer Engineering, Florida International University, Miami, FL 33174 USA

**Keywords:** Engineering, Electrical and electronic engineering

## Abstract

A circular polarized (CP) pentaband antenna based on the aperture-in-aperture (AIA) concept is presented for CubeSat applications. This AIA consists of five different bands ranging from L-band to Ka-band. Four different antennas, each operating at a specific frequency band, namely 12 GHz, 18.5 GHz, 26 GHz, and 32 GHz, were incorporated into an L-band (viz. 1.5 GHz) antenna. Notably, the five antennas can operate simultaneously for a CubeSat downlink operation with a frequency ratio of 21.3:1. The antenna structure shows a realized gain of 5–10 dBi with good CP bandwidth (< 3 dB) across the overall operational frequency range. That is, the realized gain of L-band (1.5 GHz), X-band (12.5 GHz), K-band1 (18.5 GHz), K-band2 (26 GHz), and Ka-bands (32 GHz) are 5.05 dBi, 8.21 dBi, 7.33 dBi, 7.97 dBi, and 8.56 dBi. A high impedance surface (HIS) is incorporated with the Ka-band antenna to mitigate the ripples in the radiation pattern created by the interference of surface waves. A prototype was fabricated and tested. The measurement data agrees well with the simulation.

## Introduction

In recent years, CubeSats have revolutionized satellite communication, as they are inexpensive, lightweight, and easy to launch, making them ideal for various applications, including remote sensing, navigation, and deep space scientific explorations, among others. Typically, the size of CubeSat is defined by a multiple of 1 cubic unit (1U). Notably, 1U corresponds to L × W × H = 10 cm × 10 cm × 10 cm. Due to its size limitation, a Cubesat has limited payload capacity, which means it can only carry small instruments or experiments. This restricts the size and weight of the payload to a few kilograms or even grams, depending on the CubeSat’s size. CubeSat radios are typically required to be of low size, weight, and power (SWaP) requirements. As such, antennas and radio frequency (RF) front ends must be very compact and lightweight for the designated frequency band, which is challenging to achieve^1^. Several lightweight, low profile and compact antennas were designed for CubeSat applications^2–5^. However, antennas with different frequency bands are needed to communicate with ground station and global positioning and sensing^1^. For example, UHF and L band antennas are reliable due to their low atmospheric absorption to establish initial communication with the ground station. However, these low-frequency band antennas are unsuitable for faster communication with high data rates. Nowadays, satellite and deep space communications require operating at high frequencies (viz*.* X-band, K-band, and Ka-band) that support larger bandwidth, improved signal penetration, smaller antenna size, lower levels of interference, and higher data rates^6–8^ (Supplementary Information).

Wideband and ultra-wideband (UWB) antenna technologies have become prominent solutions to harness more of the available bandwidth, especially for cellular applications. Among different types of UWB antenna configurations, a balanced antipodal Vivaldi antenna (BAVA) or tightly coupled dipole antenna (TCDA) is well-known for ultra-wideband operation^9–11^. However, their gain is often not constant, and their efficiency is comparatively low, taking many significant dips throughout the entire band. Alternatively, higher efficiency can be achieved using aperture-shared antennas that consist of integrating different band antennas onto a single substrate^12–28^. This aperture-shared concept, also known as aperture-in-aperture (AIA), allows the optimization of the gain at each band separately. Compared to UWB antennas, much higher aperture efficiency can be achieved using the AIA concept^19,20,29^. Further, AIA enables the creation of multiple spatial channels, improving the system’s data rate.

Other performance metrics of an AIA antenna are the frequency ratio, isolation, and aperture reuse efficiency. These metrics greatly depend on the design approach used. The antenna elements are alternately placed in one approach and use different feeding positions^12–14^. These antennas suffer from a low impedance bandwidth (< 10%) and low aperture reuse efficiency. Another approach employs the stacked method to place the higher band antenna on top of the lower band antenna, which effectively provides high aperture reuse efficiency^15–22^. However, the isolation among the different bands could be better for this type of shared-aperture antenna. A third approach is to insert a higher-band antenna into a lower band. This approach provides an optimized trade-off between isolation and aperture reuse efficiency^24–28^. This antenna requires several stacked substrates with aperture-coupled feeding from various stack layers to achieve multiband operation, adding complexity to the design. However, existing investigations have mainly focused on dual-band and tri-band aperture-shared antennas. To our knowledge, the development of pentaband antennas using a single planar substrate with significantly high-frequency ratios has yet to be explored. Yet, designing aperture-shared pentaband antennas on a single substrate of uniform thickness is challenging and time-intensive, particularly when considering additional design criteria such as polarization diversity and high gain.

**Figure 1:**
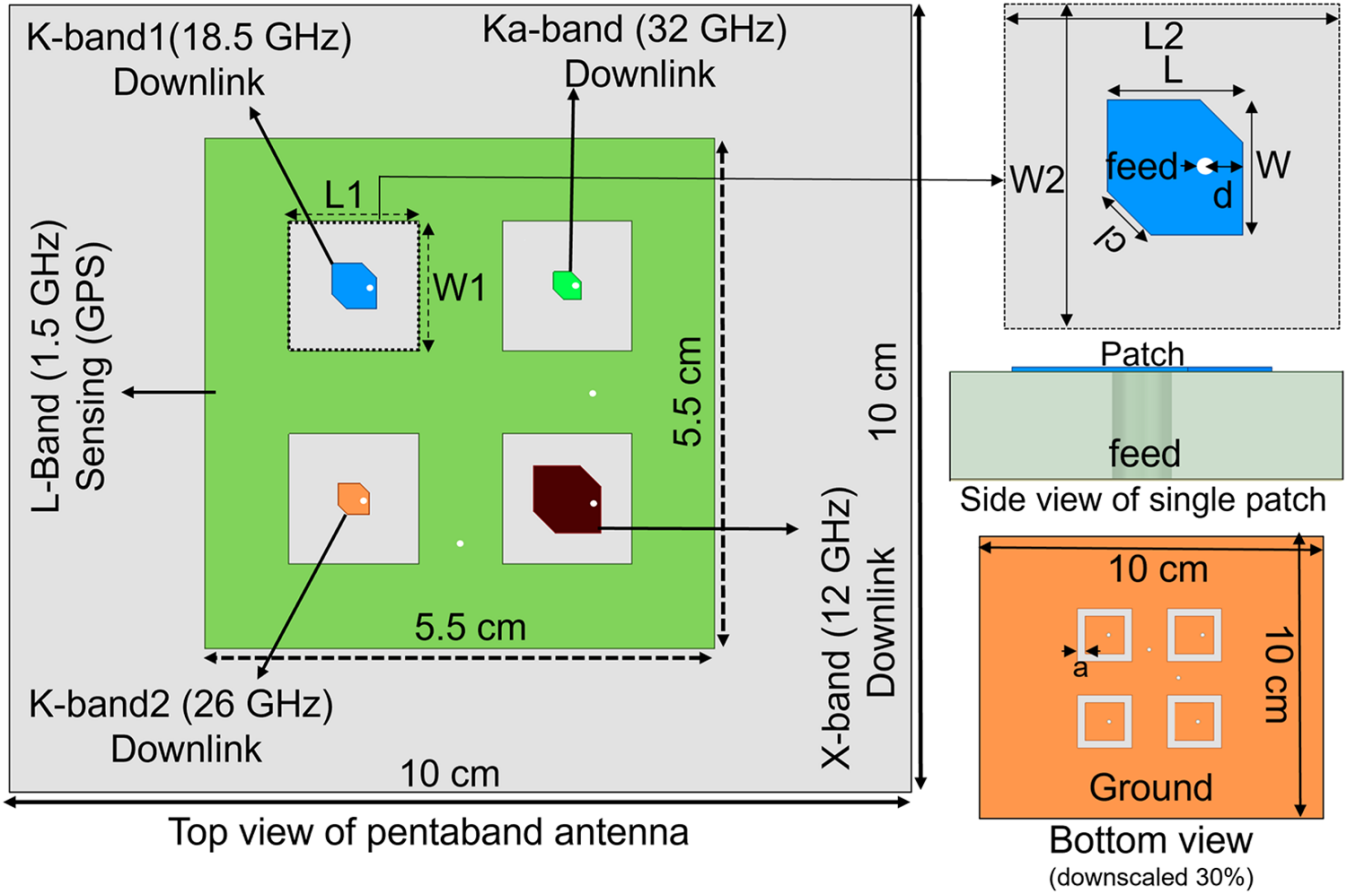
Top view and side view of the pentaband antenna.

In this paper, we present an AIA antenna for 1U CubeSats, designed to target five widely spaced frequency bands of the spectrum, namely L(1.5 GHz), X (12 GHz), K (18.5 and 26 GHz), and Ka (32 GHz) with maximum frequency ratio 21.3. The design consists of a square-shaped L-band antenna with four symmetrically etched quad-gaps, as depicted in Fig. 1. The four different band antennas are inserted into this quad-gap of L-band antenna. These antennas are designed to achieve left-hand circular polarization (LHCP) with corner truncation. They have separate ground planes to reduce the surface wave and increase the isolation. The L-band antenna has two orthogonal feeding positions to switch between linear and circular polarization when needed. In the meantime, the other four bands (12 GHz, 18.5 GHz, 26 GHz, and 32 GHz) have only single feed and corner perturbation for LHCP. In addition, the Ka-band (32 GHz) antenna is integrated with a high-impedance surface (HIS) to minimize the surface wave created from the finite discontinuity of the ground plane. Our design brings forward the following novelties: (1) Integration of L, X, K (two bands), and Ka-band antenna in the single aperture using the same substrate for the first time; (2) Separate ground plane for all antenna helps to increase the isolation (> 35 dB); (3) A high-frequency ratio (> 21); (4) Integration of a HIS for 32 GHz antenna to reduce the ripples in the radiation pattern; 5) Low power consumption and smaller size of the aperture providing an excellent option for CubeSat.

## Results

We present an aperture-shared pentaband antenna, as shown in Fig. 1. The antenna operates at the L(1.5 GHz), X (12 GHz), K (18.5 and 26 GHz), and Ka(32 GHz) bands simultaneously. The substrate is RT/Duroid 5880 (*ε*_*r*_ = 2.2 and tan*δ* = 0.009), with a thickness of 0.79 mm. The unit antenna consists of one L-band patch, one X-band patch, two K-band patches, and on Ka-band patch inside the L-band antenna. The four-square-size slots were etched from the inside of the L-band antenna, and the four different bands of the antenna were integrated into that gap. The slot dimension is 14 mm × 14 mm (L1 × W1). The overall dimension of the pentaband antenna is 10 cm × 10 cm. The L-band antenna was designed with two orthogonal feed positions to realize the circular polarization. The other band antennas employed truncated patches to realize the LHCP. The ground of all antennas was separated by symmetrically etching the copper from the ground plane of the L-band antenna with dimensions of 10 mm × 10 mm (L2 × W2). Each ground plane (X to Ka-band) is separated by 2 mm, which is depicted by "a" in Fig. 1. The presented antenna is suitable for the 1U CubeSat that will fit in the side of the CubeSat (10 cm × 10 cm), eliminating the need for a deployable wing.

The wavelength difference between maximum and minimum frequency (Ka-band (32 GHz, *λ* = 9.3 mm) and L-band (1.5 GHz, *λ* = 200 mm)) is more than 21 times (9.3 mm × 21.5 = 200 mm). Designing this AIA antenna using the same substrate and thickness is critical as the wavelength difference is too high. For that, the full-wave simulation needs to run for a long time to optimize the design. The optimized design parameters for each band antenna are shown in Table 1.

**Table 1:** Optimized parameters of the pentaband antenna.

Antenna	L (mm)	W (mm)	cl (mm)	d (mm)
X-band (12 GHz)	7.26	7.26	2.34	1.54
K-band1 (18.5 GHz)	4.85	4.85	1.55	1.02
K-band2 (26 GHz)	3.38	3.38	1.09	0.715
Ka-band (32 GHz)	3	3	1.1	0.62

**Figure 2:**
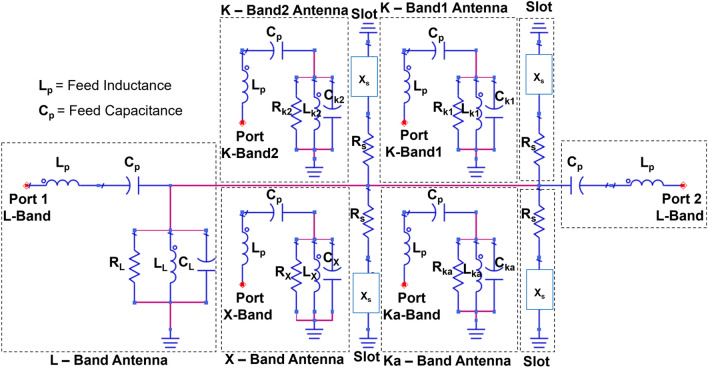
Equivalent circuit model of the pentaband antenna.

Figure 2 shows the equivalent circuit or the presented pentaband antenna; when modeling the equivalent circuit, it is essential to consider feed inductance and capacitance^30^. The feed inductance and capacitance are represented by L_*P*_ and C_*P*_, respectively. A microstrip patch antenna can be modeled as a parallel RLC circuit, where R_*L*_, C_*L*_, and L_*L*_ represent the resistance, capacitance, and inductance of the L-Band antenna. The L-Band antenna features four quad gaps or slots that allow the integration of four additional bands. Each slot can be modeled as a series of resistance and reactance, denoted by Rs and Xs^31^. Similarly, the X-Band, K-Band1, K-Band2, and Ka-Band antennas can be modeled as RLC circuits, as shown in Fig. 2. These four bands have separate ground planes. R_*X*_, C_*X*_, and L_*X*_ are the resistance, capacitance, and inductance of the X-Band antenna. Likewise, R_*K*1_, C_*K*1_, and L_*K*1_ represent the resistance, capacitance, and inductance of the K-Band1 antenna; R_*K*2_, C_*K*2_, and L_*K*2_ represent those of the K-Band2 antenna; and R_*Ka*_, C_*Ka*_, and L_*Ka*_ represent those of the Ka-Band antenna.

**Figure 3:**
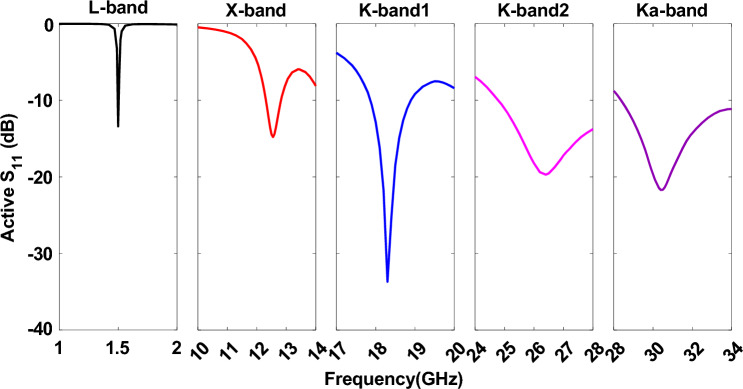
Simulated active *S*_11_ of the pentaband antenna shows an excellent match for the designated frequency band.

Figure 3 shows the *S*_11_ of pentaband antenna. The L-band antenna resonates at 1.5 GHz with a bandwidth of 1.5%. The X-band, K-band1, and K-band2 antennas have a comparatively higher bandwidth and a strong resonance near 12.5 GHz, 18.5, and 26 GHz. In contrast, the Ka-band antenna operates from 29 to 34 GHz. All antennas except the L-band antenna show wide bandwidth as these antennas have a corner perturbation and higher substrate height over the free space wavelength ratio (h/*λ*_0_). The antenna’s bandwidth is inversely proportional to the patch antenna’s quality factor (Q = Energy Stored/Power Loss). Lower Q results in higher bandwidth and vice versa. The Q factor increases with a higher dielectric constant and decreases with a thicker substrate. Therefore, a low dielectric constant and a thick substrate are desirable for high bandwidth^32,33^. In this design, we used the lowest dielectric constant from Rogers (*ε*_*r*_ = 2.2) due to its superior performance and low loss tangent over a wide frequency range. However, we selected a substrate thickness of h = 0.79 mm as a compromise between the lowest (1.5 GHz) and highest frequency (32 GHz) antenna characteristics while considering the space and weight constraints of the CubeSat. The 1.5 GHz antenna’s h/*λ*_0_ ratio is 0.00395, corresponding to a bandwidth of just over 1.3%. As frequency increases, the h/*λ*_0_ ratio also increases (since *λ*_0_ decreases with increasing frequency), thereby lowering the Q factor for the X-band (12 GHz), Ka-band (18 and 26 GHz), and K-band (32 GHz) antennas, which in turn increases their bandwidth up to 5%.

**Figure 4:**
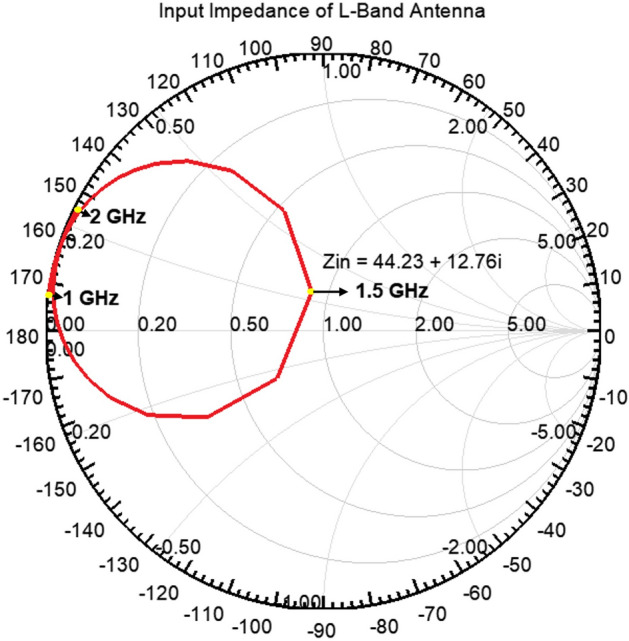
Smith chart showing the input impedance of L-band antenna.

The L-Band antenna design is particularly critical among these five different antenna bands due to its miniaturized nature. The calculated dimensions for the L-Band antenna, considering the specified dielectric (*ε*_*r*_ = 2.2, height = 0.79 mm), are 79 mm (width), and 67 mm (length). We reduced the width to optimize the integration of other antenna bands, creating a square-shaped antenna with dimensions of 55 mm × 55 mm. This modification involved cutting four symmetrical slots inside the L-Band antenna. The square shape was selected to facilitate circular polarization through two orthogonal feeds. Figure 4 shows the input impedance of the pentaband antenna in the Smith chart. The L-band antenna is highly inductive till 1.5 GHz and highly capacitive after 1.51 GHz. The input impedance of the L-band antenna is 44.23 + 12.76i, corresponding to the *S*_11_ value of around − 17 dB at 1.5 GHz.

**Figure 5:**
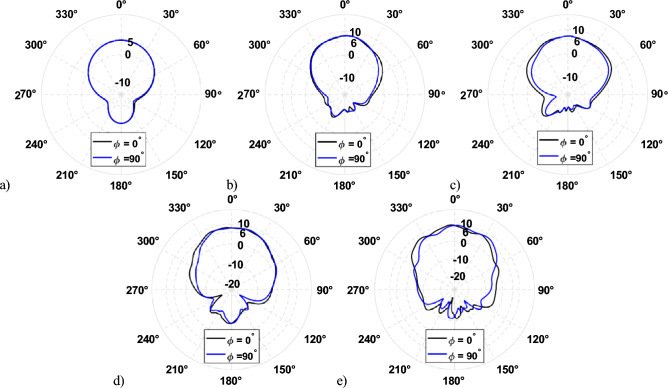
Simulated realized gain at (**a**) 1.5 GHz, (**b**) 12 GHz, (**c**) 18.5 GHz, (**d**) 26 GHz, and (**e**) 32 GHz.

The realized gain of the L-band antenna is 5.05 dBi for both the E-plane and H-plane, as shown in Fig. 5. The realized gain of X-band, K-band1, and K-band2 are 8.21 dBi, 7.33 dBi, and 7.97 dBi at 12.5, 18.5, and 26 GHz, respectively, as shown in Fig. 5b–d. However, the realized gain at 32 GHz is 8.56 dBi, and the pattern is distorted in both the E-plane and the H-plane, as shown in Fig. 5e. This is because of the interference of surface waves at 32 GHz from the finite ground plane. The mitigation of surface waves is discussed in the following section.

### High impedance surface implementation for Ka-band

**Figure 6:**
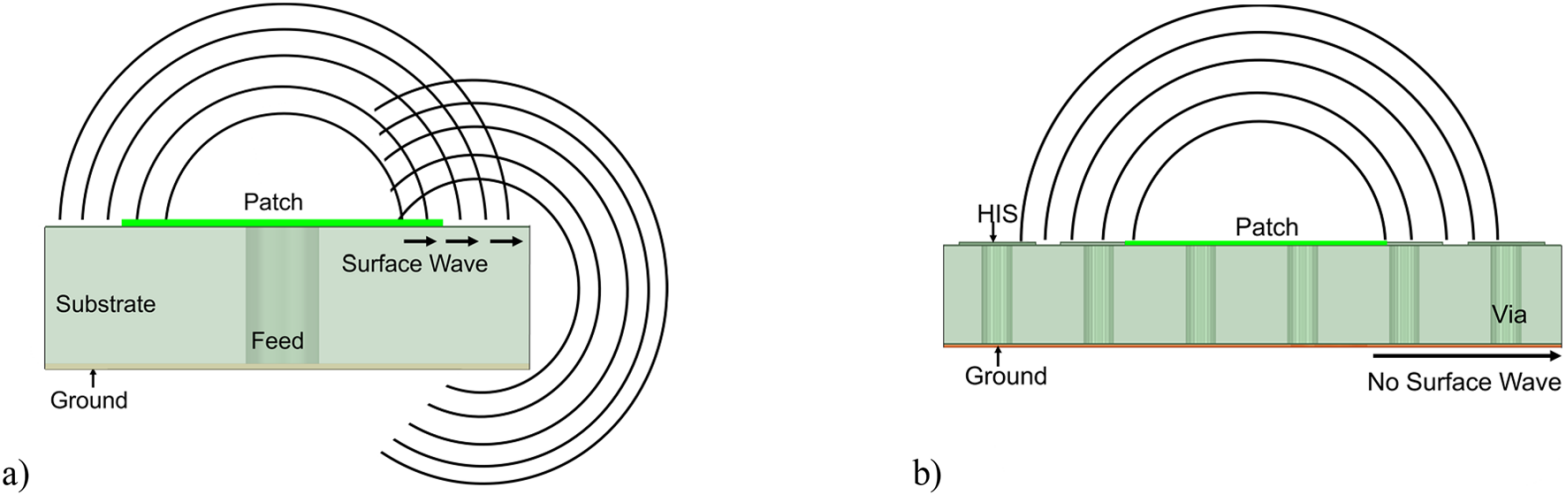
(**a**) Patch antenna on a finite ground plane creates a surface wave that distorts the radiation pattern due to interference. (**b**) Patch antenna with high impedance surface mitigates the surface wave and restores the radiation pattern.

A flat metal sheet often presents a convenient medium for surface waves to propagate^34^. The surface wave propagates electro-magnetic (EM) waves between the air interface and the metal plate. These waves radiate when there are surface discontinuities. The pentaband antenna has a finite ground plane, discontinuities, and cutting edges along the axis. Hence, the surface wave travels to the edge of the ground plane, radiates vertically, and creates interference with the radiated field of the antenna, as shown in Fig. 6a. This interference creates ripples in the radiation pattern. These ripples are dominant and more visible for the Ka-band antenna shown in Fig. 5e. The energy carried by surface waves depends on the substrate height to wavelength ratio (h/*λ*_0_); as this ratio increases, so does the power carried by the surface waves^35^. This ratio is negligible for low frequencies (L-band), making the effect of surface waves on the radiation pattern minimal. However, as frequency increases, the impact becomes more noticeable. The radiation pattern is affected in the X-band and Ka-band, resulting in less smooth patterns than the L-band antenna. The h/*λ*_0_ ratio is highest for the K-band (32 GHz) antenna, where the dominant effect of surface waves significantly degrades the radiation pattern.

**Figure 7:**
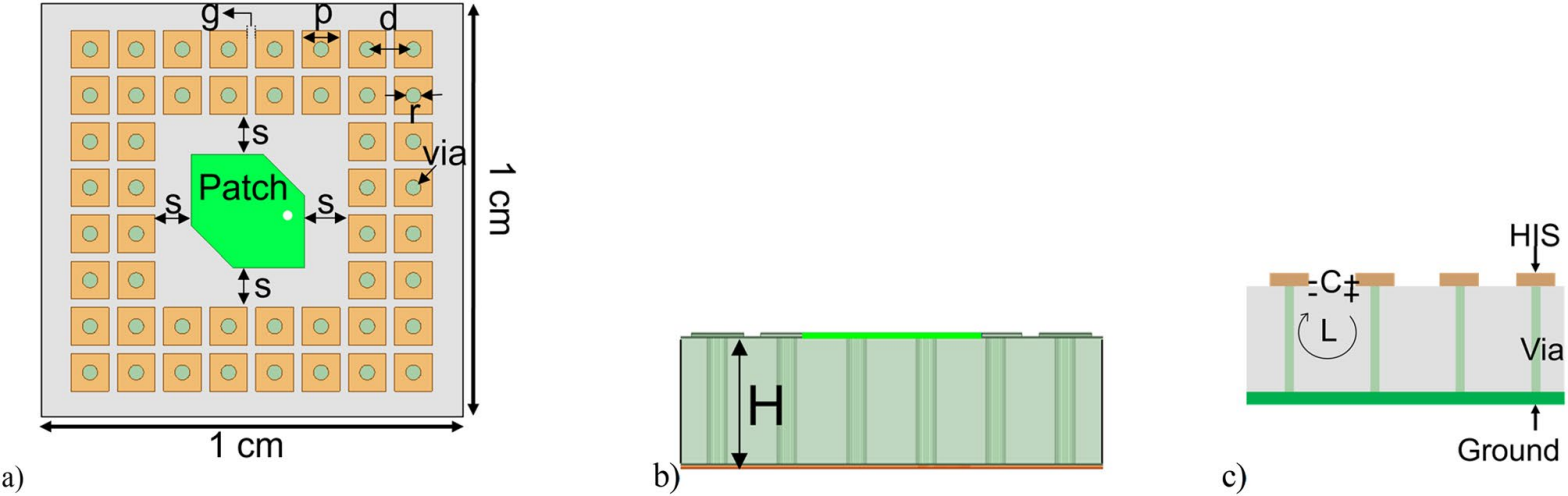
(**a**) Ka-band antenna with high impedance surface. (**b**) Side view. (**c**) Inductance and capacitance in high impedance surfaces.

To mitigate the surface wave from the finite ground plane, a new EM structure was presented^36^. This structure is widely known as a high-impedance surface (HIS). The presence of HIS helps to remove the surface wave, and thus, it prevents interference so that ripples in the radiation pattern can be minimized, as shown in Fig. 6b. HIS is a two-dimensional lattice arranged as a mushroom-like structure protruding from the lower ground plane, as shown in Fig. 7a and b. The top surface is a square-shaped patch arranged in a planar structure. The bottom surface is the ground plane shorted with the top surface using metal vias. This HIS is very small compared to the wavelength, and it can be modeled as a lumped element of capacitor and inductor, as shown in Fig. 7c. Capacitance is formed due to the gap of the top patches, and inductance is formed due to the loop between the two patches through metal via connecting the top and bottom ground plane^34^. The formula for the inductance and capacitance can be written as follows^36^.

1 $${\text{L}} = \mu_{0} {\text{H}}$$

2 $$C = \varepsilon_{0} p(\varepsilon_{r} + \varepsilon_{0} )\cos \;{\text{h}}^{ - 1} (d/g)/\pi$$

Here, *L* and *C* denote the inductance and capacitance of HIS, respectively. *ε*_0_ and *µ*_0_ are the permittivity and permeability of the free space, respectively. *ε*_*r*_ is the permittivity of substrate (*ε*_*r*_ = 2.2). The width of the square patch is *p*, the center-to-center distance of the adjacent patch is *d*, *g* is the gap between two adjacent patches, and *H* is the height of substrate as shown in Fig. 7a and b. The resonant frequency (*f*) and surface impedance (*Z*) can be written as follows.

3 $$f = {1}/{2}\pi \surd LC$$

4 $$Z = j\omega L/({1} - j\omega^{{2}} LC)$$

To design the HIS for a specified frequency of 32 GHz, we begin by calculating the inductance *L* using 1, as the height of the substrate (*H*) is fixed, allowing for a straightforward calculation. Next, we determine the capacitance *C* using 3 by substituting the desired frequency (32 GHz) and the inductance value obtained in the first step. With the required capacitance known, we refer to Fig. 7a to find and optimize the values of *p*, *d*, and *g* to match the calculated capacitance. Finally, we create vias to connect the HIS with the ground plane, ensuring that the HIS operates effectively at the specified frequency.

**Figure 8:**
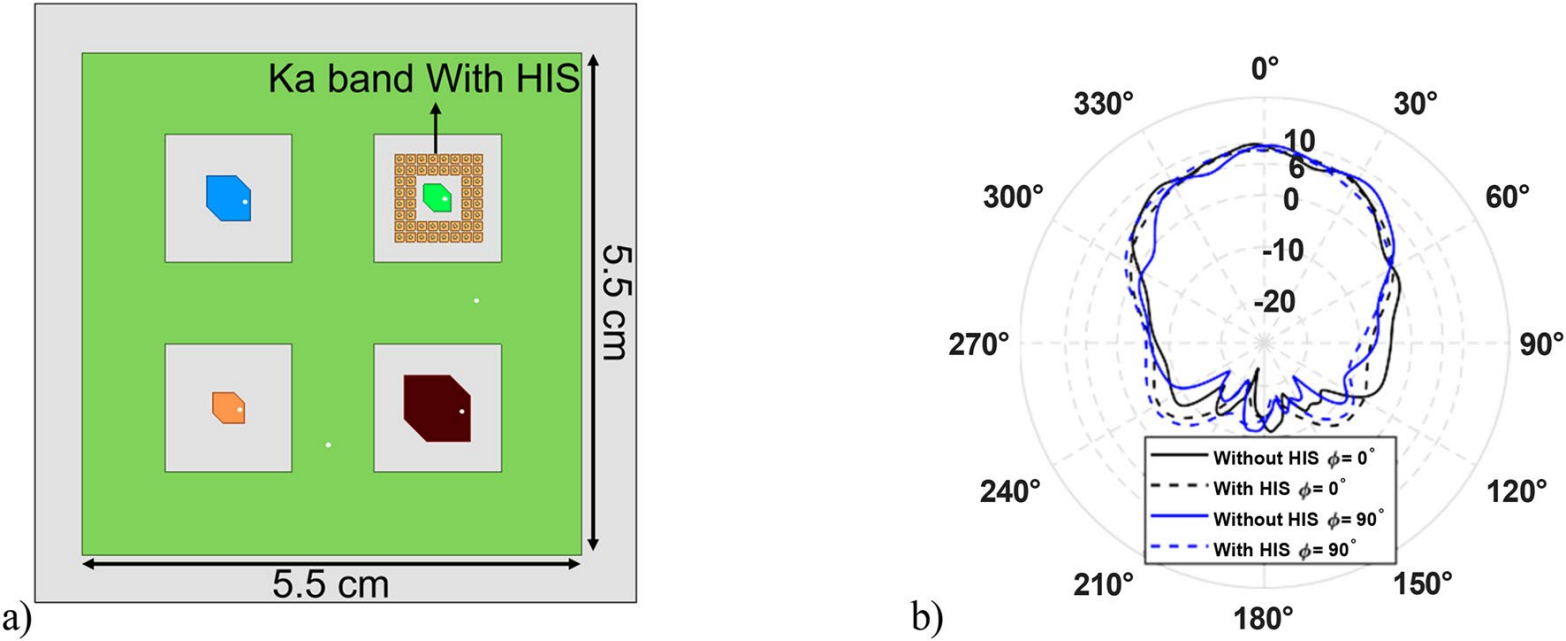
(**a**) Pentaband antenna with integrated high impedance surface at Ka-band. (**b**) Simulation result of the beam pattern at 32 GHz without and with HIS showing a smooth pattern with no ripples.

**Figure 9:**
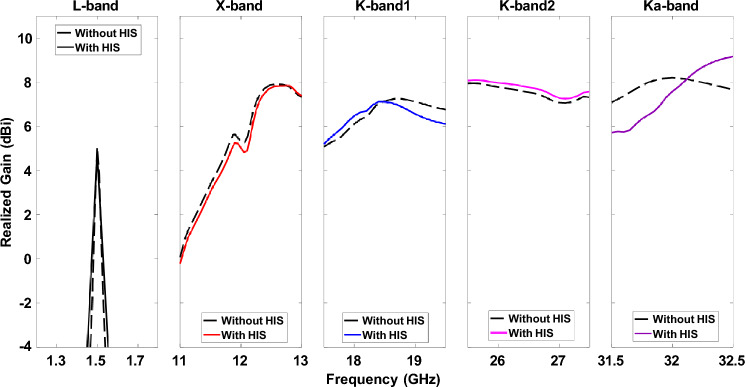
Simulated realized gain at five different frequency bands with and without HIS.

**Figure 10:**
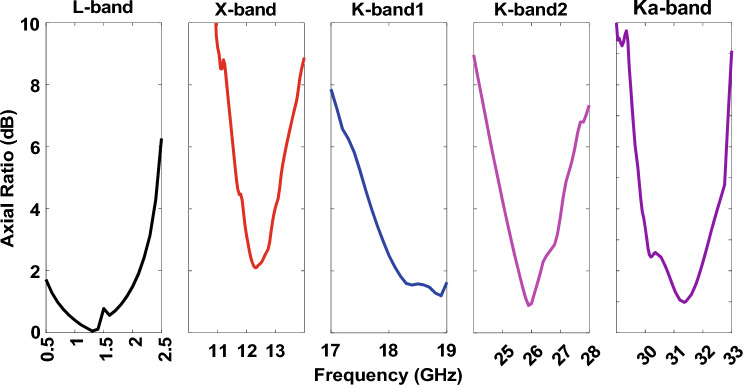
Simulated axial ratio of pentaband antenna.

**Figure 11:**
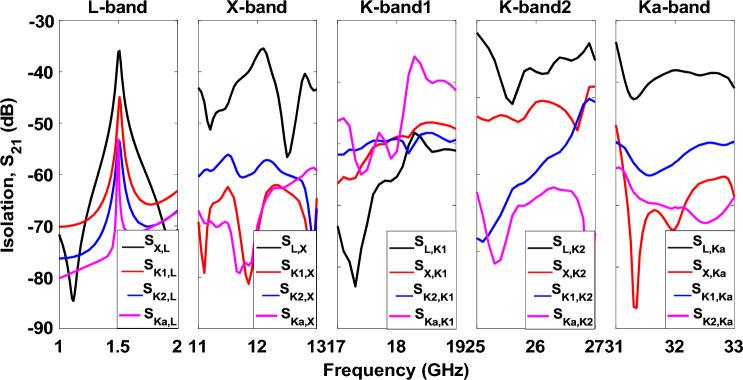
Simulated isolation among all five antennas showing higher than 35 dB.

According to the analytical 1 and 2, *p*, *d*, and *g* values are 1 mm, 1.145 mm, and 0.145 mm, respectively. However, in full-wave simulation, the abovementioned variables must be slightly adjusted, with *d* and *g* being 1.226 mm and 0.226 mm. The surface inductance and capacitance are calculated as 0.99 nH and 24.85 fF. Therefore, the surface impedance can be computed using 3 that is − 4*.*5 × 10^17^i. The resonant frequency of the HIS structure is near 32 GHz, calculated using 4. The gap between the patch antenna and HIS is 1 mm (s = 1 mm). The integrated structure of HIS for Ka-band (32 GHz) is shown in Fig. 8a. The other band antennas are unchanged, and full wave simulation was performed again. The new radiation pattern for the 32 GHz antenna is shown in Fig. 8b. As expected, the ripples of the radiation pattern are removed due to the successful mitigation of surface wave using HIS as compared to Fig. 5e. The frequency Vs. Realized gain and the frequency versus axial ratio is shown in FigS. 9 and 10, respectively. It is worth noting that the isolation among the antenna elements is higher than 35 dB for the desired frequency band, as shown in Fig. 11.

### Fabrication and assembly

**Figure 12:**
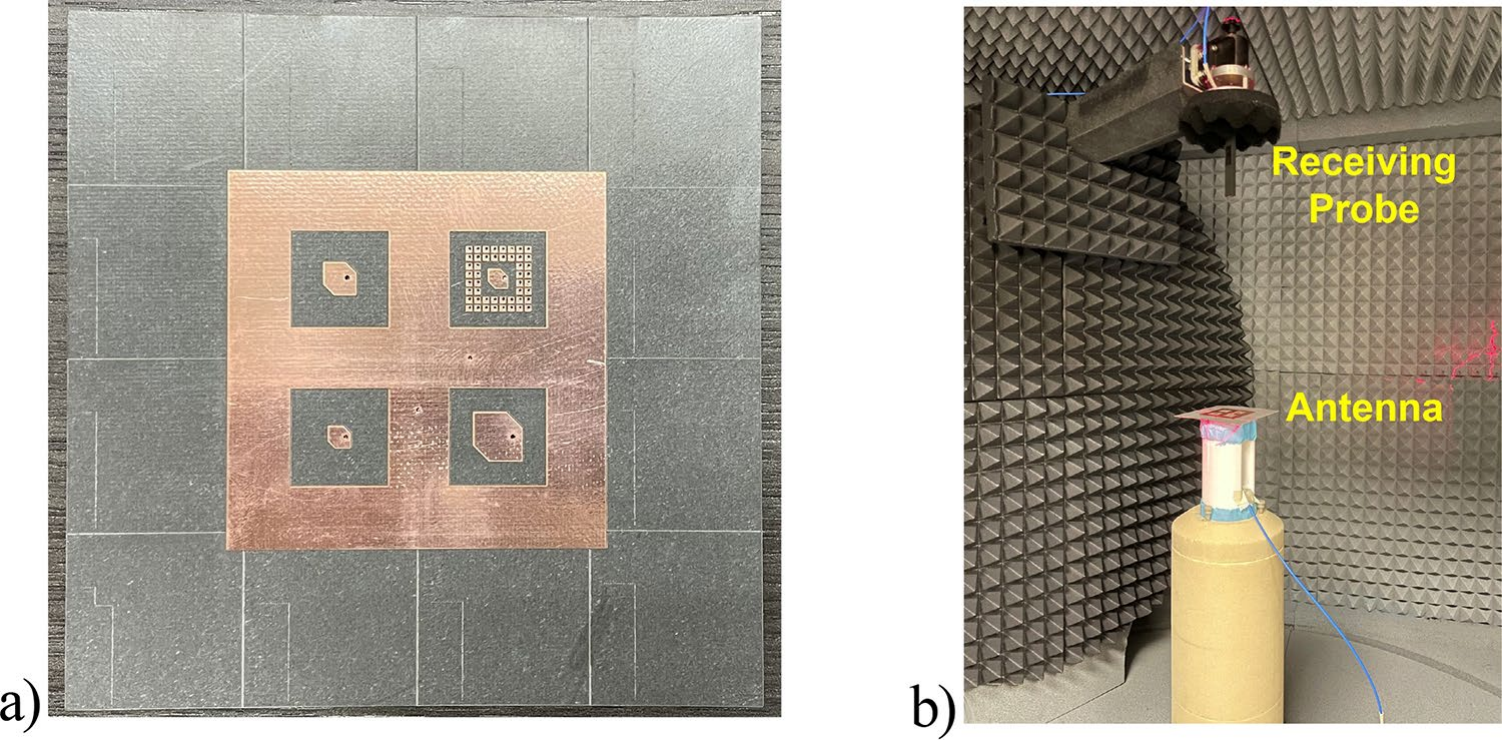
(**a**) Fabricated prototype of pentaband antenna. (**b**) Measurement setup of pentaband antenna.

A prototype of the pentaband antenna is fabricated using a standard laser machine, as shown in Fig. 12a. An extrusion method uses Ag paste under a microscope to create the HIS around the ka-band (32 GHz) antenna. The SMA connector was used to connect the L-band antenna, whereas an SMPS connector and SMPS to 2.4 mm adapter were used to connect the other four bands antenna. The SMPS was selected for the upper band due to the narrow spacing between the antenna elements.

## Discussion

**Figure 13:**
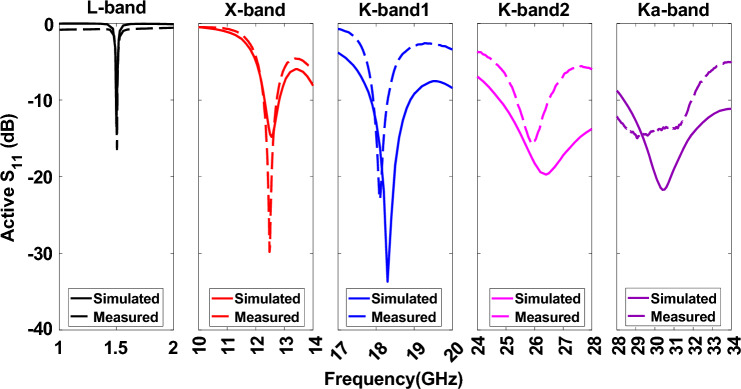
Simulated and measured active S_11_ of pentaband antenna.

**Figure 14:**
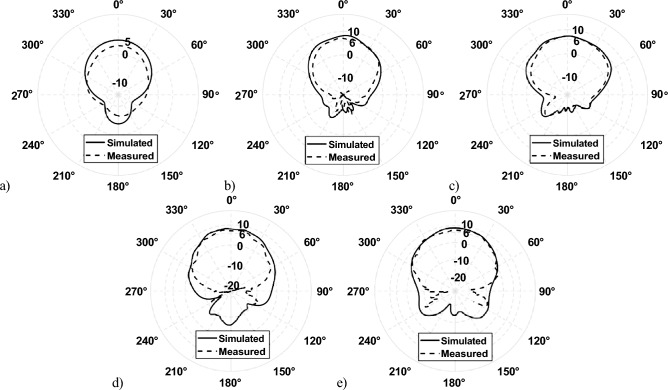
Simulated and measured realized gain for *φ* = 0° at (**a**) 1.5 GHz, (**b**) 12 GHz, (**c**) 18.5 GHz, (**d**) 26 GHz, and (**e**) 32 GHz.

**Figure 15:**
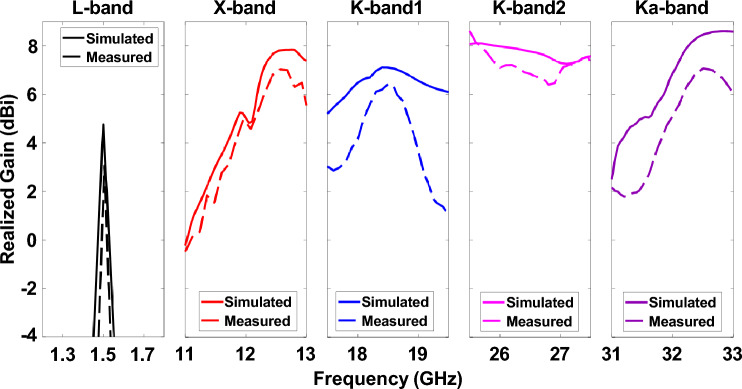
Simulated and measured realized gain of the pentaband antenna.

Figure 12b shows the measurement setup of the pentaband antenna in an anechoic chamber. The simulated and measured S_11_ re shown in Fig. 13. The measured S_11_ perfectly aligns with the L-band and X-band. However, a slight discrepancy exists between simulated and measurement data for K-band1, K-band2, and Ka-band antenna. The slight discrepancy between simulated and measured data is because of fabrication in accuracy. The measured realized gain of L-band, X-band, K-band1, K-band2, and Ka-band antenna are 3.06 dBi, 7.03 dBi, 5.83 dBi, 7.08 dBi, and 7.18 dBi, respectively, as shown in Fig. 14; the simulated realized gains are 5.05 dBi, 8.21 dBi, 7.33 dBi, 7.97 dBi, and 8.56 dBi, respectively. The minor difference between simulated and measured data stems from inaccuracies in fabrication. In contrast, the measured realized gain of the Ka-band antenna with HIS agrees with the simulated result, showing the effectiveness of HIS in suppressing the surface wave and presenting a ripple-free radiation pattern as shown in Fig. 14e. Figure 15 shows the simulated and measured realized gain. The measured gain is slightly lower than the simulated gain, but the realized gain curve pattern shows symmetry. The properties of the previously reported antenna and our presented antenna are summarised in the Table 2. Among them, our presented antenna shows a superior frequency ratio with the lowest thickness and high isolation. For instance, our design shows a much higher frequency ratio and much lower thickness compared to its close counterpart of a dual-band antenna in^37^. In^37^, a dual-band microstrip patch antenna designed to cover the S and X bands was developed. The design incorporates two dielectric stacks placed on top of the driven patch to enhance gain by forming a Fabry–Perot Resonator Cavity Antenna (FPRA). However, this approach results in increased antenna thickness (0.137*λ* ) and overall volume, posing integration challenges on the side of a 1U CubeSat due to its stringent maximum weight constraints. In contrast, our presented antenna exhibits a lightweight and compact structure with the lowest thickness (0.003*λ*) available, facilitating pentaband operation with a high-frequency ratio (21.3) and isolation (> 35 dB). This design ensures smooth operation for 1U CubeSats, and its compact form allows for easy mounting on the side of a 1U CubeSat.

**Table 2:** Performance comparison of our antenna with previously reported aperture shared antenna.

References	Aperture type	Frequency (GHz), low/high	Gain (dBi)	Max. Isolation (dB)	Thickness (*λ*)	Frequency Ratio	Polarization
18	Stacked	0.9/5.8	5.8	20	0.075	6.4	Circular
38	Stacked	0.9/2.45	5.8	–	0.026	2.7	Circular
39	Stacked	0.35/2.1	7.89	20	0.234	6	Circular
40	Insertion	1.55/29	4–10	20	0.32	18.7	Circular
37	Insertion	2.013/8.53	7.7–12.8	20	0.137	4.23	Circular
Our De-sign	Insertion	1.5/32	5–8.56	35	0.003	21.3	Circular

Thickness (*λ*) is represented according to the wavelength of the lower band; Frequency Ratio = High band/Low band.

Our design brings forward the following novelties: (1) Integration of L, X, K (two bands), and Ka-band antenna in the single aperture using the same substrate for the first time; (2) Separate ground plane for all antenna helps to increase the isolation (> 35 dB); (3) A high-frequency ratio (> 21); (4) Integration of a HIS for 32 GHz antenna to reduce the ripples in the radiation pattern by mitigating surface wave; (5) Compact and low profile antenna ensures smaller size of the aperture providing an excellent option for CubeSat.

## Conclusion

In this paper, we designed a CP pentaband antenna based on the AIA concept presented for the CubeSat application. This AIA consists of five different antenna bands, from L-band to Ka-band. Four different frequency bands, such as 12 GHz, 18.5 GHz, 26 GHz, and 32 GHz antennas, were incorporated into the L-band antenna, and they can operate simultaneously for CubeSat downlink operation. A quad square-shaped slot was etched in the L-band antenna, and four different band antennas were placed. The antenna shows good realized gain (5–10 dBic) with good CP bandwidth (< 3 dB) for the operational frequency range. This shared aperture’s maximum to minimum-frequency ratio is around 21, which is very challenging. The Ka-band antenna is incorporated with an HIS to mitigate the ripples in the radiation pattern created by the interference of surface waves. The measurement data agrees well with the simulation, which shows that this antenna can be a great candidate for the multi-frequency operation of the CubeSat antenna.

## Method

Full-wave simulation software ANSYS-HFSS was used to design the pentaband antenna. The design involved incorporating four different bands of antenna into a single L-band antenna, and the calculations of high impedance surface for the Ka-band antenna were done using 1 2 3 and 4. A prototype was fabricated and tested to validate the full-wave simulation results. The pentaband antenna prototype is manufactured using a conventional laser machine. To implement the HIS around the Ka-band (32 GHz) antenna, an extrusion technique is employed to create vias using Silver (Ag) paste under the guidance of a microscope machine.

### Supplementary Information

Supplementary Information.

## Data Availability

All data generated or analysed during this study are included in this published article [and its supplementary information files].
