# Supplementary material for: A shared-aperture pentaband antenna with high impedance surface for CubeSat application
Source: Sci Rep. 2024 Jul 12;14:16146. doi: 10.1038/s41598-024-66632-7 (PMC11245614; doi:10.1038/s41598-024-66632-7)
Supplement: Supplementary file 1 — Supplementary Information. [file 41598_2024_66632_MOESM1_ESM.pdf]

Supplementary Material

(A Shared-Aperture Pentaband Antenna with High Impedance Surface for CubeSat Application)

Md Nazim Uddin[1] **and Elias A. Alwan**[1]

[1]Department of Electrical and Computer Engineering, Florida International University, Miami, FL 33174, USA
[*]muddi027@fiu.edu

1 Design of Pentaband Antenna and High Impedance Surface Implementation

As discussed in the result section, the unit antenna consists of one L-band patch, one X-band patch, two K-band patches, and one Ka-band patch inside the L-band antenna. The four-square-size slots were etched from the inside of the L-band antenna, and the four different bands of the antenna were integrated into that gap as shown in Fig. S1. The design of the pentaband antenna into a single substrate is critical and the optimized dimension is provided in Table S1. Due to surface wave propagation, the Ka-band (32 GHz) antenna has ripples, and a high-impedance surface (HIS) was integrated around the Ka-band antenna and the design parameters are shown in Fig. S2. The equations (1), (2), (3), and (4) were used to calculate the parameters of HIS are shown in the manuscript and the optimized value is presented in Table 2s. Table 3s and Fig. S3 shows the active S_{11} of simulated and measured pentaband antenna. The realized gain of pentaband antenna is shown in Table 4s and Fig. S4.

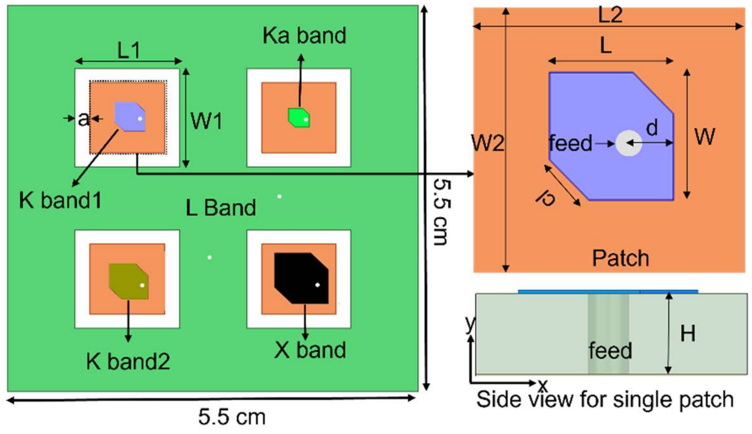

Figure S1. Top view and side view of the pentaband antenna

Antenna	L (mm)	W (mm)	cl (mm)	d (mm)	L1 × W1 (mm × mm)	L2 × W2 (mm × mm)	a (mm)	H (mm)
X-band (12 GHz) ‘	7.26	7.26	2.34	1.54	14 × 14	10 × 10	2	0.79
K-band1 (18.5 GHz)	4.85	4.85	1.55	1.02				
K-band2 (26 GHz)	3.38	3.38	1.09	0.715				
Ka-band (32 GHz)	3	3	1.1	0.62				

Table 1s. Optimized parameters of the pentaband antenna.

Design Parameters	Value (mm)	Calculated	Value
p	1	Inductance (L)	0.99 nH
d	1.226	Capacitance (C)	24.85 fF
g	0.226	Impedance (|Z|)	4.5×10^{17}
r	0.2	Resonant Frequency (f)	32 GHz
s	1	-	-

Table 2s. Optimized parameters of HIS for Ka-band antenna as shown in Fig. 2s.

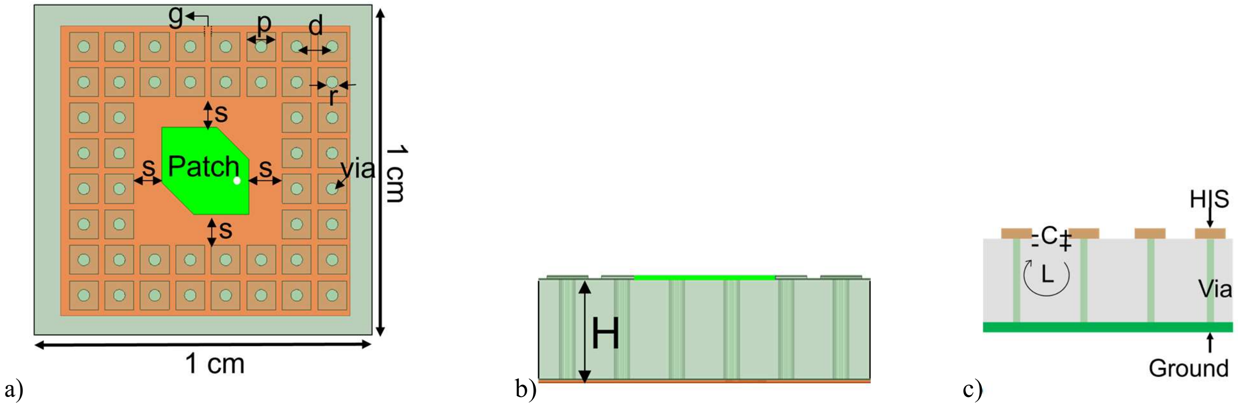

Figure S2. a) Ka-band antenna with high impedance surface. b) Side view. c) Inductance and capacitance in high impedancesurfaces.

L -Band			X-Band			K-Band1		
Freq [GHz]	Simulated (S(1,1))	Measured (S(1,1))	Freq [GHz]	Simulated (S(1,1))	Measured (S(1,1))	Freq [GHz]	Simulated (S(1,1))	Measured (S(1,1))
1	-0.021931	-0.820226	10	-0.474206	-0.481706	17	-3.793331	-0.657039
1.1	-0.017426	-0.794653	10.5	-0.696850	-0.499893	17.5	-6.090301	-1.83406
1.2	-0.017377	-0.783041	11	-1.099399	-0.685099	18.1	-16.16150	-21.6154
1.3	-0.02598	-0.761425	12	-4.864999	-3.9290001	18.3	-33.68521	-12.0217
1.4	-0.080204	-0.756598	12.5	-14.46057	-29.803646	18.7	-12.62969	-4.27738
1.5	-16.63508	-16.40675	13	-7.678708	-6.1732216	19	-9.172268	-2.93901
1.6	-0.104393	-0.799056	13.5	-5.997733	-4.6192465	19.5	-7.51077	-2.65036
1.7	-0.051561	-0.694877	14	-8.112886	-5.8640513	20	-8.417590	-3.29677
1.8	-0.054950	-0.647382						
1.9	-0.073956	-0.606940						
2	-0.107854	-0.568329						

K -Band2			Ka-Band		
Freq [GHz]	Simulated (S(1,1))	Measured (S(1,1))	Freq [GHz]	Simulated (S(1,1))	Measured (S(1,1))
24	-6.929552	-3.5155	28	-12.72207	-11.955
24.5	-8.709092	-4.5583	28.5	-15.72393	-13.582
25	-11.05799	-6.4752	29	-19.66167	-14.441
25.5	-14.36522	-10.931	29.5	-21.67442	-14.470
26	-18.23570	-15.602	30	-18.97706	-14.252
26.4	-19.70730	-11.480	30.5	-16.29920	-13.569
26.5	-19.53861	-10.372	31	-14.27734	-13.691
27	-17.18542	-7.1612	31.5	-12.93887	-12.802
27.5	-15.12462	-5.7143	32	-11.91788	-9.9330
28	-13.7645	-5.8947	32.5	-11.30590	-7.6044
-	-	-	33	-11.13227	-6.1541
-	-	-	33.5	-11.16554	-5.3386
-	-	-	34	-11.27909	-5.0538

Table 3s. Dataset of simulated and measured active S_{11} of pentaband antenna.

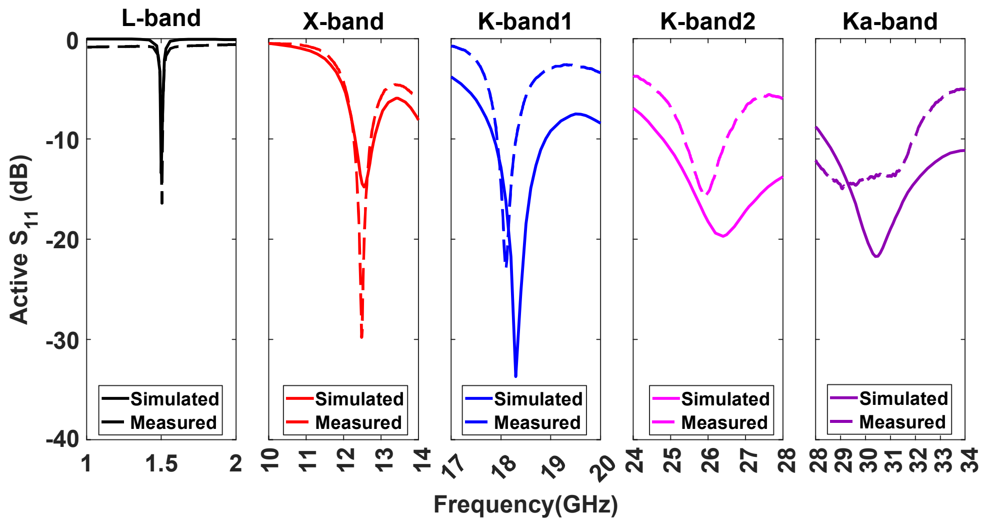

Figure S3. Simulated and measured active S_{11} of pentaband antenna.

L -Band			X-Band			K-Band1		
Freq [GHz]	Simulated Realized gain (dBi)	Measured Realized gain (dBi)	Freq [GHz]	Simulated Realized gain (dBi)	Measured Realized gain (dBi)	Freq [GHz]	Simulated Realized gain (dBi)	Measured Realized gain (dBi)
1	-32.4881	-36.333	11	-0.21974	-0.96616	17.5	5.203476	2.87011
1.1	-28.3248	-33.151	11.3	1.87461	1.344085	17.7	5.701024	3.225065
1.2	-24.3054	-29.39	11.5	3.042435	1.040255	18	6.485446	4.165938
1.3	-19.8234	-21.7751	11.7	4.037225	2.626941	18.2	6.701482	5.58847
1.4	-13.2429	-15.2078	11.9	5.252533	4.465249	18.5	7.109264	6.427259
1.5	4.761949	3.067233	12	5.023402	4.084662	18.7	6.975984	5.850662
1.6	-11.6458	-14.259	12.3	7.124347	5.6336	19	6.558557	3.644924
1.7	-17.0946	-19.0305	12.5	7.768668	6.531971	19.2	6.325686	2.061695
1.8	-20.0018	-21.5984	12.7	7.836683	5.813219	19.5	6.103575	1.143575
1.9	-21.8572	-23.6849	13	7.373861	4.594434	-	-	-
2	-23.137	-24.5034	-	-	-	-	-	-

K -Band2			Ka-Band		
Freq [GHz]	Simulated Realized gain (dBi)	Measured Realized gain (dBi)	Freq [GHz]	Simulated Realized gain (dBi)	Measured Realized gain (dBi)
25.5	8.077545	8.6138	31	2.507974	2.17313
25.7	8.102621	7.9742	31.2	4.253992	1.79206
26	7.976918	7.1081	31.5	5.020777	2.23114
26.2	7.914854	7.2115	31.7	5.408035	3.4191
26.5	7.770793	6.8334	32	6.885579	5.1032
26.7	7.653595	6.651	32.2	7.820642	6.1854
27	7.290797	6.9719	32.5	8.470828	7.0759
27.2	7.290791	7.2007	32.7	8.585298	6.9017
27.5	7.580467	7.2425	33	8.581599	6.0593

Table 4s. Dataset of simulated and measured realized gain of pentaband antenna.

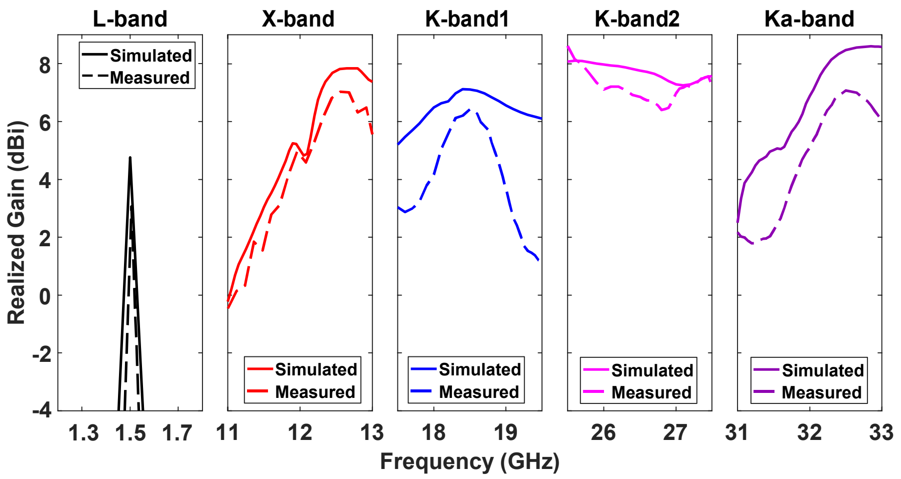

Figure S4. Simulated and measured realized gain of the pentaband antenna.
